# Supplementary material for: Curated and harmonised transcriptomics datasets of interstitial lung diseases
Source: Data Brief. 2025 Oct 14;63:112139. doi: 10.1016/j.dib.2025.112139 (PMC12581653; doi:10.1016/j.dib.2025.112139)
Supplement: Supplementary file 1 [file mmc1.zip › Supplementary_material/RNA-seq/fastQC reports/GSE184316/TRIMMED_SRR15926807_fastqc.html]

TRIMMED\_SRR15926807.fastq.gz FastQC Report 

FastQC Report

Sat 3 Dec 2022  
TRIMMED\_SRR15926807.fastq.gz

## Summary

- Basic Statistics
- Per base sequence quality
- Per sequence quality scores
- Per base sequence content
- Per sequence GC content
- Per base N content
- Sequence Length Distribution
- Sequence Duplication Levels
- Overrepresented sequences
- Adapter Content

## Basic Statistics

| Measure | Value |
| --- | --- |
| Filename | TRIMMED\_SRR15926807.fastq.gz |
| File type | Conventional base calls |
| Encoding | Sanger / Illumina 1.9 |
| Total Sequences | 7077223 |
| Sequences flagged as poor quality | 0 |
| Sequence length | 35-371 |
| %GC | 50 |

## Per base sequence quality

## Per sequence quality scores

## Per base sequence content

## Per sequence GC content

## Per base N content

## Sequence Length Distribution

## Sequence Duplication Levels

## Overrepresented sequences

| Sequence | Count | Percentage | Possible Source |
| --- | --- | --- | --- |
| CAGTGGGGCCACGAGCTGAGTGCGTCCTGTCACTCCACTCCCATGTCCCT | 121468 | 1.7163229136626046 | No Hit |
| CACCTCCCCGAAGCTCAGGGAGAGCCCTGTTAGGGCCGCCTCTGGCCCTA | 112534 | 1.5900869592494118 | No Hit |
| AGGCTACACACTGAGGACTCTGTTCCTCCCCTTTCCGCCTAGGGGAAAGT | 99260 | 1.4025275167957827 | No Hit |
| GGGAAGCGGGGAATGTCTACGTGCGTATGCACGTGGCACTCTCTGCCCGA | 84448 | 1.1932363866448747 | No Hit |
| TTCAGCCCTGATCAAGACATGAGGGAGGCAGGGGCTCAGCTGAAGAAGCT | 52950 | 0.7481748137652297 | No Hit |
| CCCTGGCTGCTACAGGAGAATAGCAGACAGGTATAGTTAAGAAACAACTT | 48494 | 0.6852122647541274 | No Hit |
| AGCTGCTAAGTGCTGTGTTGTCGTTCCCCCTGCTTAAAATAAAGTTGTTT | 45059 | 0.6366762782520771 | No Hit |
| TGATATGGTTCCTGGCAAGCCCATGTGTGTTGAGAGCTTCTCAGACTATC | 44145 | 0.6237616081901051 | No Hit |
| TGATGACACCCACCGCAACTGTCTGTCTCATATCACGAACAGCAAAGCGA | 40618 | 0.5739256767802852 | No Hit |
| CAATCCACTTGATCCCAACTCATCTCTCATTTATTTCGGCTTCTTTTATT | 34150 | 0.4825338978296996 | No Hit |
| TTACAAAAGCAGAATAAAAGCGAAAAGAAATGAAAATGTTACACTACATT | 31748 | 0.44859403186814945 | No Hit |
| TTTTTCCATGAGCTTAATGATGCTTTCTCTGGGCTTTTGGGGGAGGGTGT | 26882 | 0.37983825011589994 | No Hit |
| AACAAGCACAAAGCCAAGAAAACCAACGATGCCAGATACTGAATTTCTAT | 26115 | 0.36900066593916847 | No Hit |
| CAGCAGTTTACACCTGCTAGGGTGTTCAAAGGTCAGTGCTATAGAAATTC | 25936 | 0.3664714253033994 | No Hit |
| TTTTCCATGAGCTTAATGATGCTTTCTCTGGGCTTTTGGGGGAGGGTGTC | 19772 | 0.27937511648283514 | No Hit |
| ACAAGAAGTACATCAAAGATTACATGAAATCAATCAAAGGGAAACTTGAA | 15277 | 0.21586150386952624 | No Hit |
| GGCAATGAGCGGTTCCGCTGCCCTGAGGCACTCTTCCAGCCTTCCTTCCT | 14631 | 0.20673362984323088 | No Hit |
| GTCCACGTCACACTTCATGATGGAGTTGAAGGTAGTTTCGTGGATGCCAC | 13822 | 0.19530259255643068 | No Hit |
| TGTGGATGAATCTTGAACTGAGTTCCACTTGTAAACTTCTTGTTTCTTGT | 12802 | 0.18089015988333276 | No Hit |
| GTTCTGCAGCCCCTGTCATAAAAGGTTTTACTCTTTCTGGTCTCTGTTCT | 11891 | 0.16801787932922277 | No Hit |
| AGACATCAAATCCTGCAGACAAGGGGAGCCCTCAGTCTGCAGGGCTCCAT | 11528 | 0.1628887488779144 | No Hit |
| TCTTGGGTTATAATCATTGGCAATGTTGTAATCAACAACCCATGTTATTT | 10768 | 0.15215007355286106 | No Hit |
| GGAAAGGGGGCACTGACTGGTGATGCTGATATTGGAGTGCATCTGAGGCT | 10676 | 0.15085012864509145 | No Hit |
| GTCCAGGGTGGGCATTGTCAGGGAAGCTGCAGAGTTATTGAACCACTTGG | 10489 | 0.14820784932169015 | No Hit |
| GAAACCCAGACACATAGCAATTCAGGAAATTTGACTTTCCATTCTCTGCT | 10268 | 0.14508515557585228 | No Hit |
| CAATAAATAGGACACTTCTGCCAAAGGAACTCGTGCACAGCACCACAGAA | 10134 | 0.14319175755801394 | No Hit |
| CAAGGGTGACACCATTCAGCTCTACCTGGGGGCCAAGTTGTTGGACTCAC | 9896 | 0.13982885660095776 | No Hit |
| CTCTCTTTCTGGCCTGGAGGCTATCCAGCGTACTCCAAAGATTCAGGTTT | 9706 | 0.13714418776969442 | No Hit |
| GACGAGTCAGAAAGGTCACGTCCTGCTCTTGGTCCTTGTCAGTGCCATGT | 9584 | 0.13542034778330428 | No Hit |
| GGCATCAAAAGTGATGGACTGCCCATTGCTGGAGAAGACCTTCTCTCCTA | 9462 | 0.1336965077969141 | No Hit |
| CAATGGGGTACTTCAGGGTCAGGATGCCACGCTTGCTCTGGGCCTCGTCG | 9127 | 0.12896301275231825 | No Hit |
| TACCACTGCCACCTTCTCCATCGGCTCCACTGGCCTCGTGGTGTATGACT | 8929 | 0.12616530523342276 | No Hit |
| GGATCTTGGCTTTCTCTTTCCTCTTCTCCTCCAGGGTGGCTGTCACTGCC | 8500 | 0.12010360560914923 | No Hit |
| CTGACAGTGTATCTAGCTTTGGAAAACACAGGGGTCTGCCCTGTGAGCTG | 8395 | 0.11861997283397739 | No Hit |
| GTCTCAACATCTGTGTTGGGGAGAGTGGAGACAGACTGACGCGAGCAGCC | 8344 | 0.1178993512003225 | No Hit |
| CCAGTCTCGGCTCAGCCAAACACTGTCAGGGCCCCCAGCAGGGCCTTCAG | 8215 | 0.11607660236225424 | No Hit |
| CGAAGCGGTTGTACTTGCGGATGTAGTGCAGATAGTCTCGGCGGATGACA | 8053 | 0.11378756893770339 | No Hit |
| AGAGGGCTCCCAGAAGTGTGTGGCTGAGCTGGGTCCCCAGGCCGTGGGGG | 8006 | 0.11312346664786456 | No Hit |
| CTTCATGATGTAGCAGCAGGTGCCAGGGGCTGGCTTGTAGGCGATCAGCA | 7972 | 0.11264305222542796 | No Hit |
| TTGGAGTGGGAAGCGGGGAATGTCTACGTGCGTATGCACGTGGCACTCTC | 7747 | 0.10946383913577402 | No Hit |
| CGAGCCACAGCTCTGCCAGTACCCCAAGACTCAGCACTAGTCTGATGACC | 7702 | 0.10882799651784324 | No Hit |
| ATCAAAGAAGAACATGTGATCATCCAGGCCGAGTTCTATCTGAATCCTGA | 7697 | 0.10875734733807314 | No Hit |
| GTCCACGTCACACTTCATGATGGAGTTGAAGGTGGTCTCGTGGATGCCGC | 7668 | 0.10834758209540662 | No Hit |
| AGGCCCCAAAAGAAAACAGCGTCTTACTGGGGAGGGATATGTGCTTTCTC | 7650 | 0.10809324504823432 | No Hit |
| CGTGAATACCAAGACCTGCTCAATGTTAAGATGGCCCTTGACATTGAGAT | 7496 | 0.10591725031131562 | No Hit |
| GGCAATGAGCGGTTCCGGTGTCCGGAGGCGCTGTTCCAGCCTTCCTTCCT | 7453 | 0.10530966736529285 | No Hit |
| CCAGGGACTTTTTGTTGCCTGTAAAGTGCTCTGGCATTGCCTGAGGATAG | 7198 | 0.10170655919701838 | No Hit |

## Adapter Content

Produced by FastQC (version 0.11.7)
